# The Relationship between the Severity of Coronary Artery Disease and Epicardial Adipose Tissue Depends on The Left Ventricular Function

**DOI:** 10.1371/journal.pone.0048330

**Published:** 2012-11-02

**Authors:** Christina Doesch, Tim Süselbeck, Dariusch Haghi, Florian Streitner, Stefan O. Schoenberg, Martin Borggrefe, Theano Papavassiliu

**Affiliations:** 1 1^st^ Department of Medicine, University Medical Centre Mannheim, Medical Faculty Mannheim, University of Heidelberg, Heidelberg, Germany; 2 Department of Clinical Radiology and Nuclear Medicine, University Medical Center Mannheim, Medical Faculty Mannheim, University of Heidelberg, Heidelberg, Germany; Innsbruck Medical University, Austria

## Abstract

**Background:**

Epicardial adipose tissue (EAT) is an active metabolic and endocrine organ. Previous studies focusing mainly on patients with preserved left ventricular function (LVF) could show a correlation between increased amounts of EAT and the extent and activity of coronary artery disease (CAD). However, to date, there are no data available about the relationship between EAT and the severity of CAD with respect to the whole spectrum of LVF impairment. Therefore, we evaluated this relationship in patients with CAD.

**Methods:**

250 patients with CAD and 50 healthy controls underwent CMR examination to assess EAT. The severity of CAD was defined using the angiographic Gensini score (GSS).

**Results:**

The GSS ranged from 2–364. Linear regression analysis revealed a significant correlation between EAT and GSS (r = 0.177, p = 0.01). Patients with mild (GSS≤10) and moderate CAD (GSS>10−≤40) showed comparable EAT to healthy controls. However, in patients with severe CAD (GSS>40) EAT was significantly reduced (p<0.0001) compared to healthy controls. Interestingly, patients with the same GSS revealed different EAT depending on the left ventricular function (LVF). Patients with preserved LVF (LVF≥50%) showed more EAT mass compared to those with reduced LVF (LVF<50%) regardless of the GSS. In patients with preserved LVF and mild CAD, EAT was comparable to healthy controls (61.8±19.4 g vs. 62.9±14.4 g, p = 0.8). In patients with moderate CAD, EAT rose significantly to 83.1±24.9 g (p = 0.01) and started to decline to 66.4±23.6 g in patients with severe CAD (p = 0.03). Contrary, in CAD patients with reduced LVF, EAT was already significantly reduced in patients with mild CAD as compared to healthy controls (p = 0.001) and showed a stepwise decline with increasing CAD severity.

**Conclusion:**

The relationship between EAT and the severity of CAD depends on LVF. These findings emphasize the multifactorial interaction between EAT and the severity of CAD.

## Introduction

Inflammation plays an integral role in the pathogenesis of atherosclerotic coronary artery disease (CAD) [1]–[3]. Therefore the interest in the epicardial adipose tissue (EAT) that is located between the myocardium and the pericardium surrounding both ventricles with variable extent and distribution patterns arouse [4]–[6]. Recent studies have shown that EAT is an endocrine and paracrine source of cytokines and chemokines involved in atherosclerosis [7]–[9]. Furthermore, an increased amount of EAT was correlated to the inflammatory burden in CAD [10]–[12]. Other studies found a relation between the EAT volume and the extent [13], [14] as well as the activity of CAD [15]. These previous studies focused mainly on patients with preserved left ventricular function (LVF). However, in prior studies of our group, we could show reduced amounts of EAT in patients with severely impaired LVF due to ischemic or dilated cardiomyopathy [16], [17] using cardiovascular magnetic resonance imaging (CMR). To date, there are no data available about the relationship between the severity of CAD and EAT with regard to LVF in patients with CAD.

Volumetric EAT measurement using CMR appeared to be a reliable and reproducible method to quantify EAT [18]. Invasive coronary angiography is the gold standard for detecting CAD. It is useful not only for the diagnosis of obstructive CAD, but also for determining the severity of CAD by stratifying the patients according to the functional significance and the degree of luminal narrowing using the Gensini score (GSS).

The aim of our study was to evaluate the relation between EAT assessed by CMR and the severity of atherosclerosis as assessed by the angiographic GSS in patients with CAD and in respect to the LVF.

**Table 1:** Demographic and baseline clinical characteristics: CAD patients and healthy controls.

	Healthy Controls	All CAD patients	p^¥^	GSS≤10	GSS>10−≤40	GSS>40	p^¶^	p*
	n = 50	n = 250		n = 40	n = 72	n = 138		
Age (yrs)	**62.8±10.8**	**64.9±9.7**	0.2	63.1±9.8	65.5±9.5	65.1±9.8	0.2	0.1
Male Sex, n (%)	**40 (80)**	**198 (79.2)**	0.9	34 (85)	59 (82)	105 (76)	0.9	0.4
Body Weight (kg)	**81.9±14.0**	**78.9±14.5**	0.04	79.6±14.4	81.1±17.4	77.6±13.3	0.7	0.2
BMI (kg/m^2^)	**27.3±5.9**	**27.0±5.9**	0.1	26.2±4.4	28.0±8.8	26.7±4.1	0.2	0.1
Cardiovascular risk factors, n (%)								
•Family history	**0 (0)**	**102 (41)**	–	10 (25)	25 (35 )	67 (49)	0.4	0.4
•Hypertension	**0 (0)**	**248 (99)**	–	40 (100)	72 (100)	136 (100)	>0.99	>0.99
•Smoking	**0 (0)**	**87 (35)**	–	15 (38)	23 (32)	49 (36)	0.7	0.8
•Hyperlipidemia	**0 (0)**	**224 (90)**	–	38 (95)	63 (88)	123 (89)	0.3	0.7
•Diabetes	**0 (0)**	**81 (32)**	–	12 (30)	25 (35)	44 (32)	0.08	0.9
NYHA functional class			–					
•NYHA class I, n(%)	**0 (0)**	**60 (24)**	–	14 (35)	22 (31)	22 (16)	0.7	0.1
•NYHA class II, n(%)	**0 (0)**	**76 (30)**	–	12 (30)	22 (31)	44 (32)	0.4	0.7
•NYHA class III, n(%)	**0 (0)**	**82 (33)**	–	11 (28)	18 (25)	54 (39)	0.8	0.1
•NYHA class IV, n(%)	**0 (0)**	**30 (12)**	–	3 (8)	10 (14)	18 (13)	0.6	0.1
Severity of CAD			–					
•1-vessel disease, n (%)	**0 (0)**	**56 (22)**	–	32 (80)	11 (15)	13 (9)	<0.0001	0.3
•2-vessel disease, n (%)	**0 (0)**	**79 (32)**	–	8 (20)	37 (51)	34 (25)	0.002	0.0002
•3-vessel disease, n (%)	**0 (0)**	**115 (46)**	–	0 (0)	24 (33)	91 (66)	0.0001	<0.0001
Atherosclerosisseverity, n (%)	**–**	**63.8±59.4**	–	4.7±3.0	24.1±9.0	101.9±55.6	<0.0001	<0.0001
hs-CRP (mg/l)	**–**	**20.0±25.8**	–	10.6±15.7	25.0±34.4	21.3±23.5	0.02	0.5
Medication, n (%)			–					
•Aspirin	**0 (0)**	**209 (84)**	–	36 (90)	60 (83)	113 (82)	0.5	0.9
•Marcumar	**0 (0)**	**59 (24)**	–	6 (15)	16 (26)	37 (27)	0.5	0.5
•Beta-blocker	**0 (0)**	**243 (97)**	–	39 (98)	70 (97)	134 (97)	>0.99	>0.99
•ACE/AT II blocker	**0 (0)**	**243 (97)**	–	38 (95)	70 (97)	134 (97)	0.6	>0.99
•Diuretics	**0 (0)**	**157 (63)**	–	23 (58)	31 (43)	103 (75)	0.2	<0.0001
•Statin	**0 (0)**	**239 (95.6)**	–	38 (95)	69 (96)	133 (96)	>0.99	>0.99
•Insulin	**0 (0)**	**42 (16.8)**	–	7 (18)	9 (13)	25 (18)	0.7	0.4
•Glucose loweringdrug	**0 (0)**	**38 (15.2)**	–	5 (13)	16 (22)	19 (14)	0.7	0.3

p^¥^ = p-value comparing all CAD patients vs healthy controls,

p^¶^ =  p-value comparing CAD patients with GSS≤10 vs CAD patients with GSS>10−≤40,

p* =  p-value comparing CAD patients with GSS>10−≤40 vs CAD patients with GSS >40.

*Abbreviations: ACE: Angiotensin-converting enzyme*, *AT II: Angiotensin II receptor blockers*, *BMI: body mass index, CAD: coronary artery disease, hs-CRP: high sensitivity C-reactive protein, kg: kilogram, LVF: left ventricular function, n: number, NYHA: New York Heart Association, yrs: years.*

**Table 2:** CMR characteristics: CAD patients and healthy controls.

		Patients with CAD						
	Healthy Controls	All CAD patients	p^¥^	GSS≤10	GSS>10−≤40	GSS>40	p^¶^	p*
	n = 50	n = 250		n = 40	n = 72	n = 138		
LVF (%)	**59.2±5.4**	**36.8±15.7**	<0.0001	42.6±13.9	43.6±15.9	31.6±14.2	0.7	<0.0001
LV-EDMI (g/m^2^)	**65.3±11.2**	**88.9±26.1**	<0.0001	79.0±20.4	85.9±24.8	93.3±27.5	0.1	0.07
LV-EDVI (ml/m^2^)	**72.3±14.3**	**115.5±45.2**	<0.0001	103.1±38.4	101.8±37.8	126.6±42.7	0.9	0.0002
LV-ESVI (ml/m^2^)	**29.6±7.7**	**78.6±46.1**	<0.0001	62.9±37.7	62.1±39.0	91.1±47.9	0.9	<0.0001
LVRI (g/ml)	**1.0±0.2**	**0.8±0.2**	0.001	0.8±0.3	0.9±0.2	0.8±0.2	0.4	0.002
LV-EDD (mm)	**50.5±5.5**	**61.8±10.9**	<0.0001	60.4±8.8	59.2±8.3	63.5±10.5	0.5	0.002
TAPSE (cm)	**2.8±0.6**	**2.4±0.7**	0.0003	2.6±0.6	2.4±0.7	2.4±0.7	0.2	0.4
RV-FS(%)	**33.0±6.1**	**28.2±8.0**	0.0001	30.3±6.9	28.3±7.8	27.5±8.2	0.2	0.5
RV-EDD (ml)	**42.7±5.6**	**42.0±7.5**	0.5	43.3±7.2	43.3±7.5	40.8±7.1	0.9	0.02
RAD (mm)	**43.8±5.8**	**45.4±7.5**	0.15	45.4±6.9	46.1±0.8	44.9±7.3	0.6	0.3
Extent of LGE expressed as percentage ofLV mass	**–**	**32.7±24.4**	**–**	24.7±21.1	26.8±22.3	40.9±23.2	0.6	<0.0001
EAT volume (ml)	**66.9±15.3**	**58.7±22.5**	0.01	61.2±19.8	67.5±26.9	53.3±19.5	0.19	<0.0001
indexed EAT volume (ml/m^2^)	**33.7±6.6**	**30.7±10.9**	0.04	31.2±8.8	35.1±12.9	28.2±9.8	0.09	<0.0001
EAT mass (g)	**62.9±14.4**	**55.2±21.2**	0.01	57.5±18.7	63.4±25.3	49.9±17.8	0.19	<0.0001
indexed EATmass (g/m^2^)	**31.8±5.6**	**28.8±10.2**	0.04	29.8±8.1	33.1±12.1	26.3±8.9	0.13	<0.0001

p^¥^ = p-value comparing all CAD patients vs healthy controls,

p^¶^ =  p-value comparing CAD patients with GSS≤10 vs CAD patients with GSS>10−≤40,

p* =  p-value comparing CAD patients with GSS>10−≤40 vs CAD patients with GSS>40.

*Abbreviations: EAT: epicardial adipose tissue*, *EDD: end-diastolic diameter*, *EDMI: end-diastolic mass index*, *EDVI: end-diastolic volume index*, *ESVI: end-systolic volume index, LV: left ventricular, LVF: left ventricular function*, *LVRI: left ventricular remodeling index*, *RAD: right atrial diameter*, *RV: right ventricular*, *RV-FS: right ventricular fractioning shortening*, *TAPSE: tricuspid annular plane systolic excursion.*

## Materials and Methods

The study was performed in accordance with federal laws and regulations, international accreditation standards and institutional policies. We obtained ethical approval of the local ethical committee, Medical Ethic Commission II, Faculty of Medicine Mannheim, University of Heidelberg, Germany. The initial approvals for this study 2011-201N-MA (Medical Ethic Commission II) have been reconfirmed on January 2011. Written informed consent was obtained from all subjects and data were analyzed anonymously.

**Figure 1:**
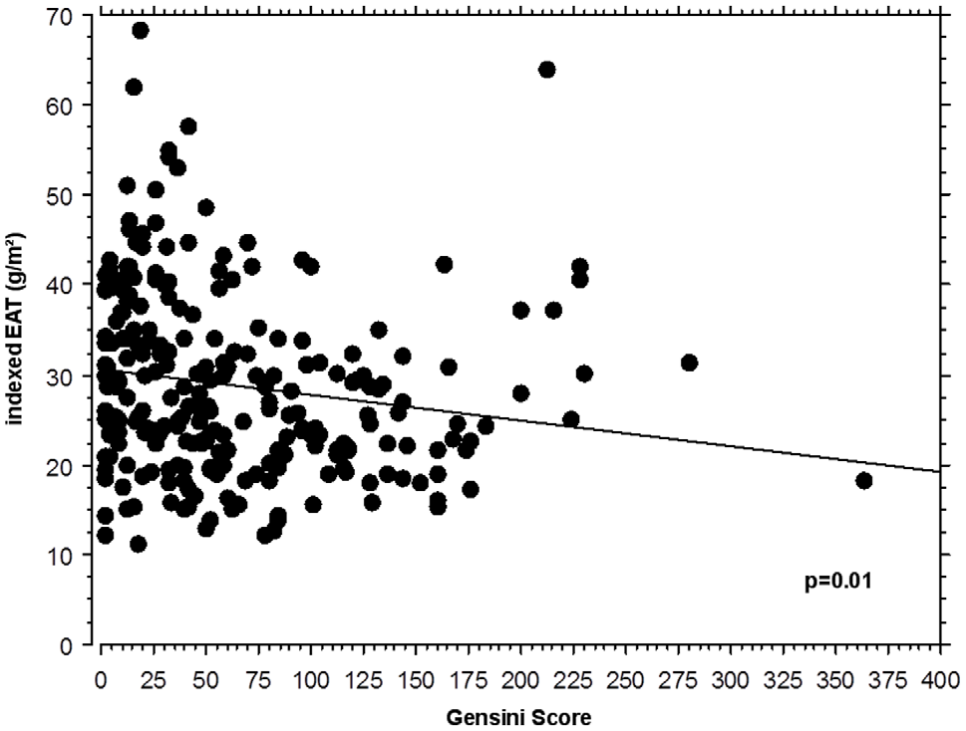
Correlation between EAT and atherosclerosis severity in all CAD patients. The linear regression analysis in all CAD patients revealed a significant correlation between indexed EAT mass and the severity of atherosclerosis as assessed by the angiographic GSS (Pearson correlation coefficient r = 0.177, associated p-value = 0.01). *Abbreviations: CAD: coronary artery disease, EAT: epicardial adipose tissue, GSS: Gensini score.*

### Study Population

300 subjects thereof 250 consecutive patients (198 (79.2%) males; mean age, 64.9±9.7 years) and 50 age and sex matched healthy volunteers were included in the study. The CAD patients presented at our hospital between January 2011 and March 2012 to undergo cardiac catheterization as part of a diagnostic evaluation for suspected or known CAD. In 181/250 CAD patients high sensitive C-reactive protein (hs-CRP) was determined as part of the routine clinical work up at the time of CMR examination. Exclusion criteria were standard contraindications to CMR examination. EAT mass was assessed in all CAD patients. Furthermore, a subgroup analysis in CAD patients with preserved LVF≥50% and reduced LVF<50% [19], [20] was performed and the EAT mass was correlated to the atherosclerosis severity.

**Figure 2:**
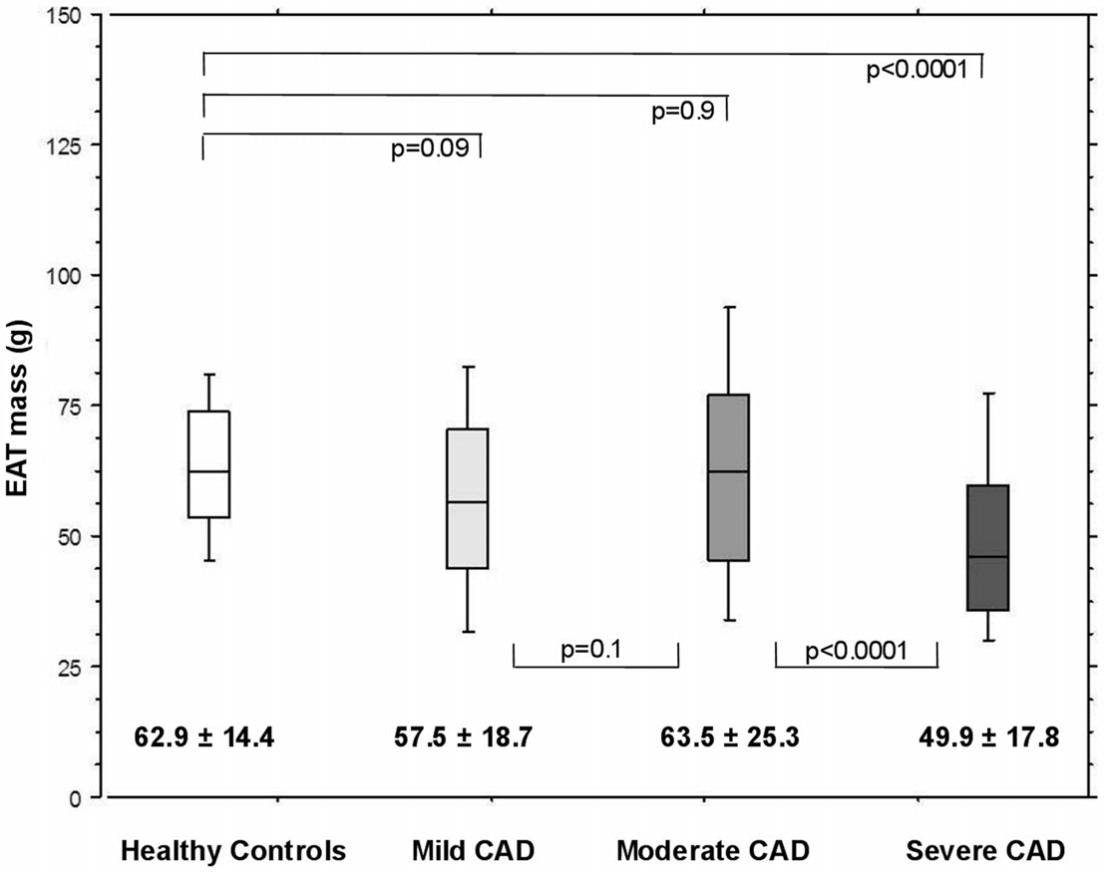
Relationship between EAT and atherosclerosis severity in the whole study population. The box plots illustrate the EAT mass in healthy controls (white box plot), patients with mild CAD (GSS≤10, light gray box plot), moderate CAD (GSS>10−≤40, dark gray box plot) and severe CAD (GSS>40, black box plot). EAT mass is given as mean ± standard deviation below the box plots, the line in the box plots indicates the median value of the data. The p-values for the comparison of the groups are also indicated. The figure shows that the EAT mass was comparable between healthy controls and CAD patients with mild and moderate CAD. However, in patients with severe CAD, a significant decrease in EAT mass compared to patients with moderate CAD and healthy controls could be detected. *Abbreviations: CAD: coronary artery disease, EAT: epicardial adipose tissue, GSS: Gensini score.*

50 age and sex matched healthy subjects served as controls and satisfied the following criteria: normal physical examination, normal blood pressure (systolic<130 mm Hg and diastolic<85 mm Hg), normal ECG findings, no history of chest pain or dyspnoea, no diabetes, no hyperlipidemia and normal 2D echocardiography and doppler examination. None of the control subjects was on medication. Exclusion criteria for healthy controls were the presence of signs or symptoms of cardiac diseases, hypertension, diabetes, smoking, or participation in competitive sports.

**Figure 3:**
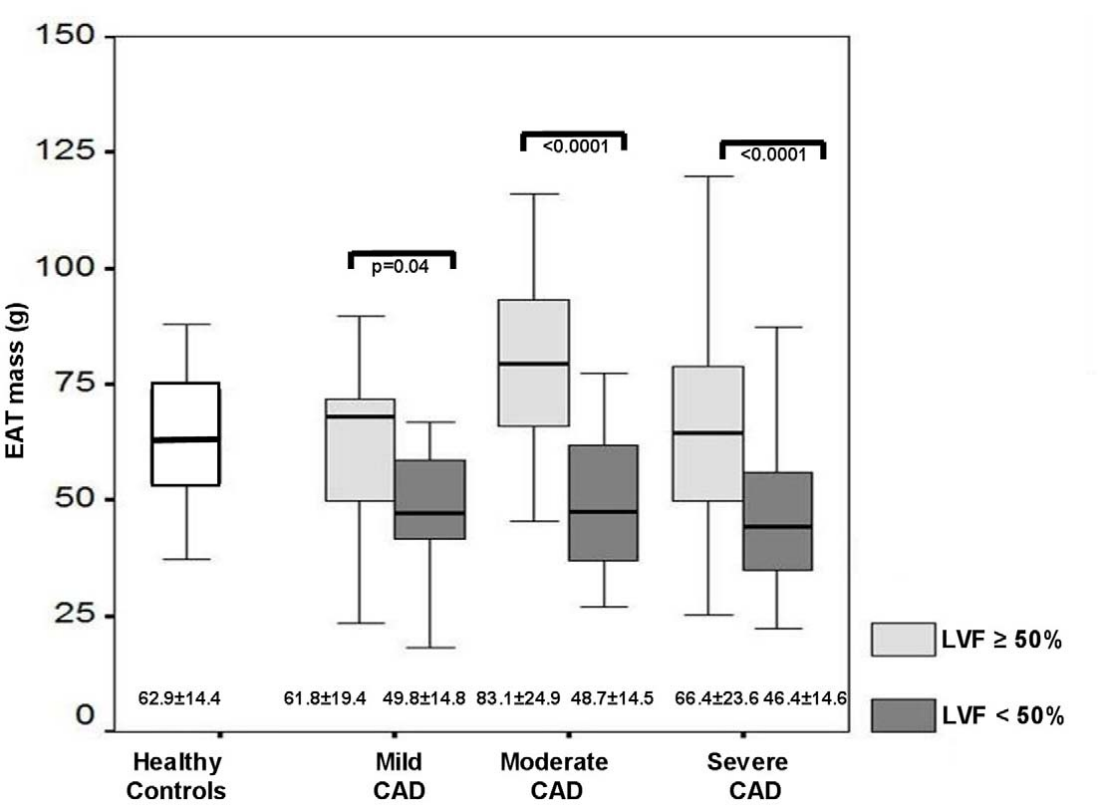
Comparison of EAT mass in patients with the same atherosclerosis severity with regard to LV function. The box plots show the EAT mass in healthy controls (white box plot) and CAD patients. The EAT mass between CAD patients with preserved LVF (LVF≥50%, light gray box plots) and reduced LVF (LVF<50%, dark gray box plots) are compared in patients with moderate, mild and severe CAD and the p-values are given. EAT mass is displayed as mean ± standard deviation below the box plots, the line in the box plots indicates the median value of the data. The figure clearly outlines that patients with preserved LVF (light gray box plots) showed significantly more EAT mass compared to those with reduced LVF (dark gray box plots) regardless of the GSS. *Abbreviations: CAD: coronary artery disease, EAT: epicardial adipose tissue, LVF: left ventricular function, GSS: Gensini score.*

All patients and volunteers underwent cardiovascular magnetic resonance imaging (CMR) examination with identical protocols except for contrast agent administration omitted in healthy controls.

**Figure 4:**
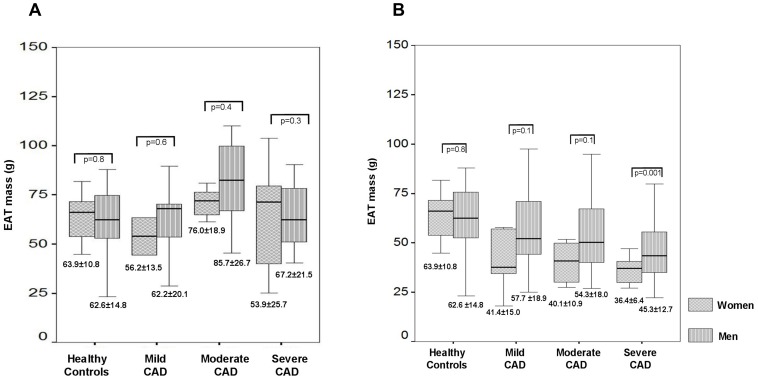
Relationship between EAT and atherosclerosis severity with regard to gender. The box plots demonstrate the relationship between EAT mass and atherosclerosis severity with regard to gender in patients with preserved LVF (LVF≥50%, Panel A) and reduced LVF (LVF<50%, Panel B). EAT mass in men is indicated by striped box plots, whereas EAT mass in women is shown by squared box plots. The p-values for the differences of the EAT mass between men and women are also indicated. EAT mass is given as mean ± standard deviation below the box plots, the line in the box plots indicates the median value of the data. In patients with preserved LVF≥50% (Panel A) and mild CAD (GSS≤10), EAT mass was comparable to healthy controls (white boxplot). With increasing atherosclerosis severity, EAT mass rose significantly in patients with moderate CAD (GSS>10−<40). However, in patients with severe atherosclerosis (GGS>40), EAT mass already started to decline. This EAT course was comparable in men and women. In patients with reduced LVF<50% (Panel B), EAT mass was significantly reduced compared to healthy controls (white boxplot) and showed a stepwise decline with increasing atherosclerosis severity in men and women. *Abbreviations: CAD: coronary artery disease, EAT: epicardial adipose tissue, LVF: left ventricular function, GSS: Gensini score.*

### Coronary Angiography and Image Interpretation

Coronary angiography (CA) was performed with a conventional angiography unit (Integris H; Philips Medical Systems). Coronary artery stenoses were imaged in the centre of the field from multiple projections. An overlap of side branches and foreshortening of relevant coronary arteries was avoided as far as possible. The severity of coronary atherosclerotic lesions was evaluated from at least two projections in all the patients. All coronary angiograms were evaluated in consensus of two board-certified cardiologists with at least 5 years experience who were blinded to the patient?clinical information. A significant stenosis was defined as a diameter stenosis of ≥50%. The extent of CAD was quantified using the number of vessels with ≥50% stenosis. The severity of atherosclerosis was determined using the GSS. As previously described [21] the GSS was calculated by assigning a severity score to each coronary stenosis according to the degree of luminal narrowing and its importance due to localization. Mild atherosclerosis was classified as a GSS ≤10, moderate atherosclerosis as a GSS >10 and ≤40 as well as severe atherosclerosis as a GSS >40 [22].

**Figure 5:**
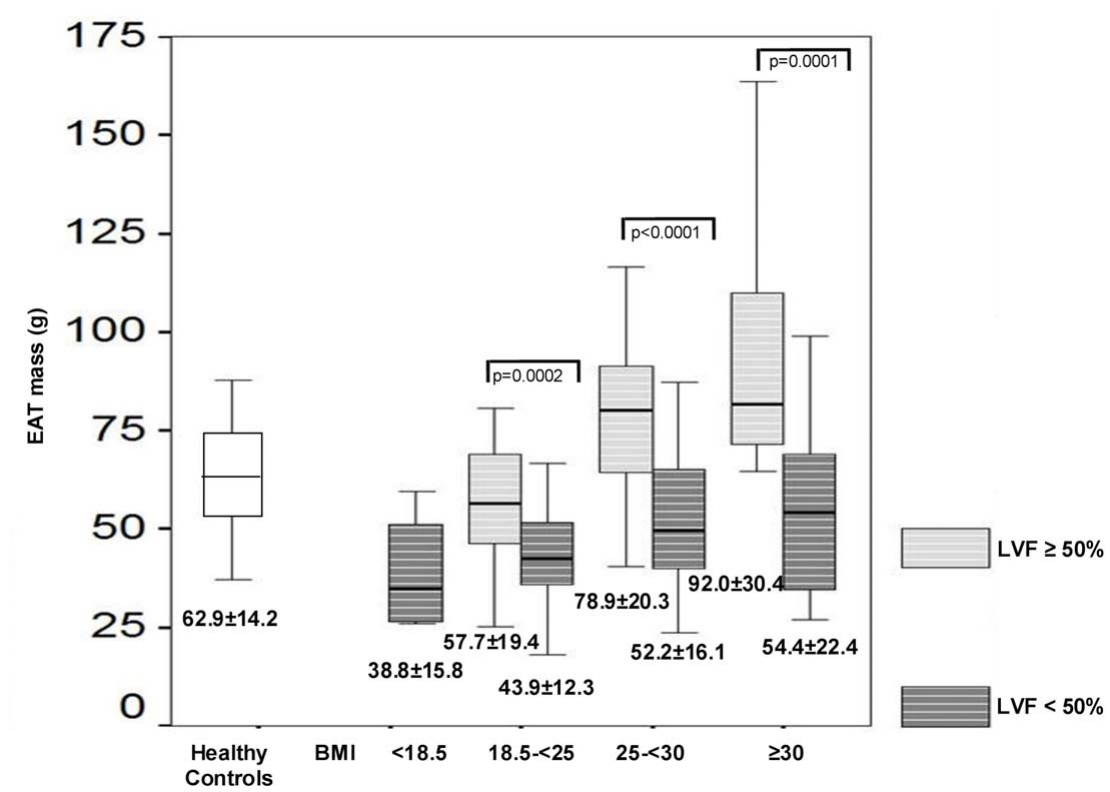
Comparison of EAT in different stages of obesity with regard to LVF. The box plots compare the EAT mass in healthy controls (white box plot) and CAD patients with preserved LVF (LVF ≥50%, light gray striped box plot) and patients with reduced LVF (LVF <50%, dark gray striped box plots) in different stages of obesity as defined by BMI. The p-values for the differences between CAD patients with preserved and reduced LVF are given. The EAT mass is depicted below the box plots as mean ± standard deviation, the line in the box plots indicates the median value of the data. In patients with CAD the EAT mass increased with augmentation of body mass index. Comparing CAD patients with the same degree of obesity, the EAT mass was dependent on LVF, revealing a significantly lower increase in EAT in CAD patients with reduced LVF compared to those with preserved LVF. *Abbreviations: BMI: body mass index, CAD: coronary artery disease, EAT: epicardial adipose tissue, LVF: left ventricular function.*

### Image Acquisition

All studies were performed using a 1.5 Tesla whole-body imaging system (Magnetom Avanto, Siemens Healthcare Sector, Erlangen, Germany). A dedicated 6-element, phased-array cardiac matrix coil in combination with two elements of the inbuilt spine matrix coil was used. Images were acquired during repeated end-expiratory breath-holds. Scout images (coronal, sagittal, and axial planes) were obtained for planning of the final double-oblique long-axis and short-axis views. To evaluate functional parameters, electrocardiogram-gated cine images were then acquired using a segmented steady-state free precession [fast imaging with steady-state precession (true-FISP)] sequence (time to echo/time of repetition 1.6/3.2 ms, temporal resolution 35 ms, in-plane spatial resolution 1.4×1.8 mm, slice thickness 8 mm, interslice gap 2 mm). Seven to 12 short-axis views covering the whole left and right ventricle were obtained. For the assessment of the EAT, we used a dark blood prepared T1-weighted multislice turbo spin-echo pulse sequence with a water suppression prepulse to obtain a transversal 4-chamber view and short-axis images in the same orientations used for the cine short-axis images. Imaging parameters were as follows: time of repetition = 800 ms, time to echo = 24 ms, slice thickness = 6 mm, interslice gap = 2 mm, and field of view = 30 to 34 cm.

### Late Gadolinium Enhancement (LGE)

Ten minutes after injection of a gadolinium-based contrast agent (Magnevist, Bayer-Schering Pharma AG, Berlin, Germany), late gadolinium enhancement (LGE) images were acquired in continuous short-axis views, the 4-chamber and the 2-chamber long axis view using an inversion recovery Turbo FLASH 2D sequence: field of view 300–340 mm, TR 9.56 ms, TE 4.38 ms, TI 200–360 ms, flip angle 25°; matrix 166×256 and slice thickness 6 mm. LGE was only considered to be present if it was also present in the same slice after swapping phase encoding, thus excluding artifacts.

### Image Analysis and Determination of Ventricular Parameters

Image analysis and quantitative analysis was performed off-line using dedicated software (ARGUS, Siemens). Each study was examined for abnormalities in the morphology of the right and left ventricle. End-diastolic and end-systolic volumes and left ventricular mass were analysed on the serial short-axis true-FISP cine loops, using manual segmentation. Stroke volumes and ejection fractions were calculated. Additionally, left- and right-ventricular diameters were measured.

On the four-chamber view, the distance between the cutting edge of the tricuspid annulus with the right ventricular (RV) free wall and the RV apex was measured in end-diastole (end-diastolic length, EDL, mm) and end-systolic length (ESL, mm). The tricuspid annular plane systolic excursion (TAPSE) was determined as the difference between EDL and ESL. The right ventricular fractional shortening (RVFS) was calculated as follows: RV−FS (%) = [(EDL−ESL)/EDL)*100] [23].

### Volumetric Assessment of the Absolute Mass of EAT

The amount of EAT was calculated by using the modified Simpson’s rule with integration over the image slices (EAT volume = ∑ [EAT area*(slice thickness + interslice gap) [18]. The contours of EAT were outlined at end diastole in the short-axis views covering the entire left and right ventricle. For EAT mass determination, the area subtended by the manual tracings was determined in each slice and multiplied by the slice thickness to yield the fat volume. Total EAT volume was obtained after the data summation of all slices. To obtain EAT mass, the volume of EAT was multiplied by the specific weight of fat (0.94 g/cm^3^). The observer was blinded to patient details.

### Extent of LGE

The extent of LGE was assessed visually by two independent experienced readers blinded to all patient details. LGE was only considered to be present if it was also present in the same slice after swapping phase encoding, thus excluding artifacts. With respect to the AHA recommendations, the myocardium was divided into 17 segments [24]. A score ranging from 0 to 4 was visually attributed to each of the 17 segments according to the transmural extent of the hyperenhancement: score 0 = 0%, 1 = 1–25%, 2 = >25%–50%, 3 = >50%–75% and 4 = >75%–100%. All these 17 scores were summed. The resulting summed score ranged in theory from 0 to 68 and was thereafter expressed as a percentage of the maximum possible score of 68 [25].

### Laboratory Measurements

High-sensitivity C-reactive protein (hs-CRP) was measured with a latex-enhanced immunonephelometric assay on a Dimension® analyzer (Dade Behring; normal range<5 mg/l).

### Statistical Analysis

Body mass index (BMI) was calculated by the common formula: BMI (kg/m^2^)  =  weight (kg)/height (m)^2^. Body surface area (BSA) was assessed by a variation of the DuBois and DuBois formula: BSA (m^2^)  =  [weight (kg)^0.425^ * height (cm)^0.725^] * 0.007184 [26]. The Kolmogorow-Smirnow-test was used to test for normality. The data are presented as mean±standard deviation (SD) for normally distributed quantitative variables and n(%) for qualitative variables. Data that are not normally distributed are given as medians and ranges. Continuous variables between two groups were analyzed by the unpaired, 2-tailed student’s t-test. The Mann-Whitney **U** test (Chi-square test) was applied for nonparametric data. Linear regression analysis was performed between EAT mass and Gensini socre in CAD patients. A p-value <0.05 was considered statistically significant.

Analysis was performed using SPSS statistical software (version 14.0, SPSS Inc., Chicago, Illinois).

## Results

Patient’s demographic and baseline clinical characteristics are shown in Table 1. The CMR characteristics are summarized in Table 2. CA was performed in all 250 patients and showed 1-vessel disease in 58 (23.2%), 2-vessel disease in 76 (30.4%) and 3-vessel disease in 116 (46.4%) patients. The GSS ranged from 2–364 with a mean of 63.8±59.4. According to the GSS, mild CAD (GSS≤10) was present in 40 (16%), moderate CAD (GSS>10−≤40) in 72 (28.2%) and severe CAD (GSS>40) in 138 (55.5%) patients. Hs-CRP in the whole CAD patient population was 20.0±25.8 mg/l. Patients with mild CAD had significantly less hs-CRP 10.6±15.7 mg/l than patients with moderate 25.0±34.4 mg/l (p = 0.02) or severe CAD 21.3±23.5 mg/l (p = 0.01).

Dividing the CAD patients according to LVF, 60 (24%) presented with preserved LVF (≥50%) and 190 (76%) with a reduced LVF (<50%). Body surface area (BSA) between patients with preserved and reduced LVF was identical 1.9±0.2, but body mass index (BMI) was diminished in patients with reduced LVF (28.4±9.2 vs 26.5±4.3, p = 0.04).

The EAT mass in all patients with CAD ranged from 18.0 g to 163.6 g with an average of 54.9±21.4 g. Linear regression analysis revealed a significant correlation between EAT mass and the GSS (r = 0.177, p = 0.01) in all CAD patients (Figure 1).

CAD patients with mild and moderate CAD showed comparable EAT mass to healthy controls. However, in patients with severe CAD, EAT mass was significantly reduced (p<0.0001) compared to those with moderate CAD (p<0.0001) and healthy controls (p<0.0001) (Figure 2).

Furthermore, our results showed that EAT was significantly different depending on the LV function when comparing patients with the same severity of CAD but different LVF (61.8±19.4 g vs 49.8±14.8 g, p = 0.04, 83.1±24.9 g vs 48.7±14.5 g, p<0.0001 and 66.4±23.6 g vs 46.4±14.6 g/m^2^, p<0.0001, respectively, Figure 3).

Therefore, we additionally analyzed the relationship of EAT to the atherosclerosis severity in patients with preserved and reduced LVF separately. Subgroup analysis of EAT mass in CAD patients with preserved LVF (LVF≥50%) showed that patients with mild CAD had a comparable EAT mass to healthy controls (61.8±19.4 g vs 62.9±14.4 g, p = 0.8). In patients with moderate CAD, EAT mass increased to 83.1±24.9 g and was therefore elevated compared to healthy controls (62.9±14.4 g, p<0.0001) and patients with mild CAD (61.8±19.4 g, p = 0.01). In patients with severe CAD and preserved LVF, the EAT mass was comparable to healthy controls (66.4±23.6 g vs 62.9±14.4 g, p = 0.5) but reduced compared to those with moderate CAD (66.4±23.6 g vs 83.1±24.9 g, p = 0.03). Looking at the hs-CRP levels in patients with preserved LVF, we found a significant positive correlation between increased EAT mass and elevated hs-CRP serum concentrations (r = 0.397, p = 0.01). Additionally, a significant positive correlation was found between increasing GSS and elevated hs-CRP serum concentrations (r = 0.463, p = 0.003).

In patients with CAD and reduced LVF (<50%), EAT mass was significantly reduced compared to healthy controls (49.8±14.8 g vs 62.9±14.4 g, p = 0.001) and showed a gradual decline in EAT mass with increasing atherosclerosis severity (48.7±14.5 g vs 46.4±14.6 g). In patients with reduced LVF, EAT (r = 0.06, p = 0.5) and GSS (r = 0.01, p = 0.9) showed no association with levels of hs-CRP.

Subgroup analysis with regard to gender showed the same EAT course for both sexes in CAD patients with preserved LVF (Figure 4A) and reduced LVF (Figure 4B). Comparing the EAT mass in CAD patients between men and women, the latter showed lower EAT mass however this difference did not reach statistical significance except in patients with severe CAD and reduced LVF.

In addition, we analyzed the proportion of obesity and its effect on EAT in our patient population. In patients with preserved LVF, body weight was normal (BMI 18.5−<25 kg/m^2^) in 21(35.0%) patients, 24(40.0%) presented with overweight (BMI 25−<30 kg/m^2^) and 15(25.0%) were obese (BMI≥30 kg/m^2^). In patients with reduced LVF, 4(2.1%) patients had a low body weight (BMI<18.5 kg/m^2^), 68(35.8%) showed a normal body weight, 83(43.7%) patients presented with overweight and 35(18.4%) were obese. The EAT mass increased with augmentation of body mass index. However, when comparing CAD patients with the same degree of obesity, once again the EAT mass was dependent on LVF. As illustrated in Figure 5 patients with reduced LVF revealed significantly lower amounts of EAT compared to those with preserved LVF.

## Discussion

The main finding of our study is that the relationship between EAT mass and the severity of CAD depends on the LVF. Therefore, patients with the same severity of CAD have significantly different amounts of EAT depending on the LVF. This observation was independent of gender. In the early stage of the disease process in absence of LVF impairment, the severity of CAD rises with increasing EAT. As soon as severe atherosclerosis is present, the EAT amount starts to decline and further decreases with the development of ischemic cardiomyopathy.

EAT is a source of various proinflammatory cytokines and chemokines with both favourable and unfavourable effects [27]. In CAD, EAT is supposed to play an integral role in the formation of atherosclerosis and disease progression [28]. Available data [29]–[32] showed a correlation between increasing EAT and progressive CAD severity. In an early echocardiographic study, Jeong JW et al. [29] showed in 203 patients with known CAD that a higher EAT thickness measured at end-diastole from the parasternal long-axis views was associated with a high GSS and therefore concluded that EAT thickness was significantly correlated with the severity of CAD. Another study by Bettencourt N et al. [30] studied the relations between EAT, abdominal visceral fat and coronary atherosclerotic burden as assessed by multislice computed tomography (MSCT) in 215 patients. They found that EAT volume positively correlated to coronary atherosclerotic burden, as assessed by coronary artery calcium (CAC) and that this correlation was shown to be independent of abdominal visceral fat. Alexopoulos N et al. [31] performed EAT volume measurement and CAC scoring in 214 consecutive patients referred for coronary computed tomography angiography (CTA). They showed a significant increase of EAT volume with the severity of luminal stenosis. Besides, they could demonstrate that EAT was an independent predictor of CAC, atherosclerotic plaques and obstructive CAD. Yerramasu A et?al. [32] performed serial CAC scans and EAT volumetry after a mean duration of 2.7±0.3 years in 270 asymptomatic diabetic patients. They could show that EAT volume measured on non-contrast computed tomography (CT) identifies individuals at increased risk of CAC progression. Besides, EAT but not intra-thoracic fat was related to the prevalence and progression of CAC burden, suggesting a paracrine local effect of this fat depot on the coronary arteries. Furthermore, Djaberi R et al. [33] explored the relation between EAT volume and coronary atherosclerosis using multislice CTA in 190 patients. They found the highest EAT volumes in patients with a CAC of 101–400 and observed a decline in EAT mass in those patients with CAC >400. Similar to these findings, our study observed in the whole study population of patients with CAD, an augmentation in EAT mass as the severity of atherosclerosis increased from mild to moderate and a significant decrease in EAT as the severity of atherosclerosis increased to severe. However, in contrast to our results, the decrease in EAT volume in patients with patients with CAC >400 did not reach statistical significance.

The studies mentioned above focused mainly on patients with preserved LVF. However, previous studies of our group [16], [17] using CMR and a very recent CT study by Khawaja T et al. [34] could show that patients with LV dysfunction revealed decreased EAT volumes compared to patients with normal LVF. To the best of our knowledge, currently no published studies are available concerning the relationship between the severity of CAD and EAT with regard to LVF in patients with CAD. Therefore, we aimed to investigate the influence of LV function impairment on the relationship between EAT and the atherosclerosis severity assessed by GSS. Thus, we compared the EAT amount between patients with the same severity of CAD but different LVF. We observed that EAT was significantly elevated in patients with preserved LVF irrespective of the CAD severity (Figure 3) indicating the multifactorial interaction between EAT and atherosclerosis.

Furthermore, we performed a subgroup analysis among CAD patients with preserved LVF and showed an initial augmentation in EAT mass with increasing atherosclerosis severity. The largest EAT amount was found in patients with a moderate atherosclerosis severity (GSS>10 and ≤40). Additionally, patients with preserved LVF showed a significant positive correlation between elevated hs-CRP serum concentrations and increased EAT mass and GSS.

These findings support the hypothesis that at the beginning of the atherosclerotic process, EAT is suggested to play a significant role in the inflammatory process. EAT secretes various proinflammatory adipokines like interleukin (IL)-1, IL-6, IL-8, IL-10, tumor necrose factor (TNF) α, leptin, macrophage chemoattractand protein 1 (MCP-1), type I plasminogen activator inhibitor (PAI-1), resistin [35] that provide a metabolic milieu that promotes atherosclerosis [4], [7], [36]. In these patients a mass-dependent mechanism could be assumed to explain the increased atherosclerosis severity coming along with elevated EAT mass. Hs-CRP is an acute-phase protein synthesized in the liver in response to the cytokine IL-6. In this context, we found in our CAD patients with preserved LVF elevated hs-CRP levels correlating with increased EAT mass. However, a causal relation between the total amount of EAT and the secretory activity could not yet be established [37]. In patients with severe atherosclerosis (GSS>40), our study already revealed a gradual decrease of the EAT mass. We hypothesize that since EAT is supplied by branches of the coronary arteries [4], severe CAD may induce a lack of nourishment and therefore result in the decline of the EAT mass.

In patients with impaired LV function, the EAT mass was reduced compared to healthy controls. In contrast to CAD patients with preserved LVF, those with reduced LV function showed a gradual reduction of EAT mass with increasing CAD severity as assessed by GSS. We speculate that in patients with severely reduced LVF, the role of EAT as a source of proatherogenic mediators does not seem to have priority. This hypothesis is supported by the fact that in contrast to CAD patients with preserved LVF, those with reduced LVF did not show an association between hs-CRP levels and EAT mass or GSS. On the contrary, with decreasing EAT mass in patients with impaired LVF, the lack of the EA?s protective properties [4], [7], [38] seem to be preponderate and therefore may indirectly influence further LV function impairment. This assumption is supported by the fact that the cardioprotetcive adipokines adrenomedullin and adiponectin have been shown to be down regulated in EAT of patients with chronic CAD but increased as soon as the hemodynamic condition improved after coronary revasularization [39], [40]. Another important factor may be the limited space in the pericardial sac. Moreover, limited epicardial fat tissue perfusion due to progressive CAD may play an important role in CAD patients since EAT derives its blood supply from the coronary arteries. Besides, reduction of EAT leads to a decreased capacity for release of free fatty acids (FFA) in times of high myocardial energy demand [41]. Finally, in patients with severe ischemic cardiomyopathy cardiac cachexia may facilitate EAT decrease.

In summary, the explanation of decreasing EAT with low LVF is still theoretical but certainly multifactorial and the exact mechanisms require further investigation.

Additionally, our results showed an identical EAT course in both genders with regard to CAD severity and LVF. In line with previous studies [18], [42] women showed a slightly lower EAT mass compared to men.

Looking at the relationship between EAT and body weight, EAT was more abundant in obese CAD patients than in lean ones. The results of previous studies investigating the association between EAT and body weight are consistent with our findings [38], [43], [44]. However, our study showed that the increase in EAT with progressive obesity is also dependent on LVF.

### Limitation

We assume that EAT changes underly a dynamic process. However, since the data were acquired at a single time point we do not have a longitudinal follow-up to confirm this hypothesis. In the future, longitudinal studies to investigate the relationship between EAT and LVF should be considered. Besides, since we did not include bypass patients, we were not able to obtain EAT biopsies in order to determine the epicardial tissue inflammation. Due to the soley descriptive nature of our study, inferences about the causal relation between EAT mass and CAD or LVF cannot be drawn. Further studies in CAD patients with different stages of LV function impairment undergoing bypass surgery are needed to further elucidate the mechanisms that regulate the EAT LVF relationship.

### Conclusion

The relationship between EAT mass and the severity of CAD depends on LVF. Patients with the same severity of CAD have different amounts of EAT depending upon the LVF impairment. This observation was independent of gender. In the early stage of the disease process, the severity of CAD rises with increasing EAT. As soon as severe atherosclerosis is present, the EAT amount starts to decline and further decreases with the development of ischemic cardiomyopathy. These findings emphasize the multifactorial nature of the relationship between EAT and the severity of CAD.
